# Sex-specific exposure prevalence of established risk factors for oesophageal adenocarcinoma

**DOI:** 10.1038/sj.bjc.6605804

**Published:** 2010-08-10

**Authors:** M Rutegård, H Nordenstedt, Y Lu, J Lagergren, P Lagergren

**Affiliations:** 1 Upper Gastrointestinal Research, Department of Molecular Medicine and Surgery, Karolinska Institutet, Norra Stationsgatan 67, Second Floor, Stockholm 17176, Sweden

**Keywords:** oesophagus, population-based, neoplasm

## Abstract

**Background:**

There is an unexplained male predominance in the incidence of oesophageal adenocarcinoma, and the sex-specific distribution of its risk factors in the general population is not known.

**Methods:**

A random sample of Swedish citizens aged 40–79 years completed a questionnaire for assessment of the prevalence of five risk factors for oesophageal adenocarcinoma: reflux symptoms, body mass index, tobacco smoking habits, socioeconomic status, and use of non-steroidal anti-inflammatory drugs (NSAIDs). Logistic regression was used to calculate odds ratios (ORs) with 95% confidence intervals (CIs) to evaluate the association of these risk factors, separately and combined, with male sex, with women as reference.

**Results:**

Among 6969 invited people, 4906 (70.4%) completed the questionnaire. Adjusted prevalence estimates showed a negative association with male sex with regard to reflux disease (OR=0.70, 95% CI=0.58–0.84), whereas overweight (OR=1.98, 95% CI=1.72–2.27) and obesity (OR=1.22, 95% CI=1.01–1.47), previous smoking (OR=1.50, 95% CI=1.30–1.72), and no NSAID use (OR=1.35, 95% CI=1.15–1.49) were positively associated.

**Conclusions:**

Exposure to some but not all established risk factors for oesophageal adenocarcinoma seems to be more common in men than in women, but the differences are small and unlikely to explain the male predominance of this tumour.

The striking male predominance of oesophageal adenocarcinoma, with a male-to-female ratio ranging from 7 : 1 to 10 : 1 (Vizcaino *et al*, 2002), remains unexplained. The rapid rise in the incidence of oesophageal adenocarcinoma in Western societies during recent decades (Brown *et al*, 2008) has been especially pronounced in men (Vizcaino *et al*, 2002). Epidemiological studies evaluating the hypothesis that sex hormones have a role, including hormonal replacement therapy (Lindblad *et al*, 2006) and reproductive factors (Lagergren and Jansson, 2005), have not provided support for oestrogen as an aetiological factor, whereas reports on anti-androgen therapy have shown mixed results (Lagergren and Nyren, 1998; Cooper *et al*, 2009). Furthermore, there seem to be no clear sex differences in the strength of the associations between known risk factors and risk of oesophageal adenocarcinoma (Hampel *et al*, 2005; Lindblad *et al*, 2005; Kubo and Corley, 2006). Thus, the explanation underlying the strong and age-specific male predominance in oesophageal adenocarcinoma remains unknown (Rutegard *et al*, 2010). The principal aetiological factors are gastro-oesophageal reflux (reflux) (Lagergren *et al*, 1999a; Shaheen and Ransohoff, 2002) and a high body mass index (BMI; Chow *et al*, 1998; Lagergren *et al*, 1999b; Kubo and Corley, 2006; Abnet *et al*, 2008), whereas tobacco smoking (Gammon *et al*, 1997; Lagergren *et al*, 2000; Freedman *et al*, 2007) and low socioeconomic status (Jansson *et al*, 2005) are weaker factors; regular use of non-steroidal anti-inflammatory drugs (NSAIDs) seems to be protective (Abnet *et al*, 2009). The sex-specific distribution of the exposure to these five factors in an unselected general population has not hitherto been estimated. We hypothesised that these risk factors for oesophageal adenocarcinoma, individually or in different combinations, are unevenly distributed between men and women. Furthermore, such sex differences might relate to pre- and post-menopausal age. To test these hypotheses, we conducted a population-based prevalence study in Sweden.

## Materials and methods

This was a population-based, cross-sectional study, with data collection during the period April–June 2008. The exposure prevalence rates of the five known aetiological factors, that is, reflux, BMI, tobacco smoking, socioeconomic status, and NSAID use, were compared between men and women in a random Swedish population sample of age 40–79 years. Random sampling and data collection were carried out by Statistics Sweden, a Swedish authority that holds the highly complete and updated nation-wide Swedish Total Population Register, which was used for this study. The sampling was performed to mimic the age and sex distribution of oesophageal and gastric adenocarcinoma according to the new cases reported to the Swedish Cancer Register in the year 2006. This provided a sample with a higher proportion of women and a slightly younger female population than had the sample been matched to oesophageal adenocarcinoma only. This age discrepancy was adjusted for in all analyses, but influenced the unadjusted prevalence, whereas all results were stratified by sex. A validated questionnaire (Lane *et al*, 2002) was sent to the selected individuals. Up to two reminding letters were sent to non-responders.

The questionnaire contained questions about the five study exposures, together with some general characteristics, including sex, age, and physical activity. Reflux was defined as heartburn or regurgitation occurring at least once a week during the last 3 months, or at least weekly use of anti-reflux medication during the same time period, a definition commonly used in epidemiological research. In addition, a reflux variable based on the Montreal definition (Vakil *et al*, 2006), including both frequency and severity of symptoms, was evaluated. Current BMI value was calculated as body weight in kilograms divided by the square of body height in metres. Cutoffs for BMI were predetermined and based on the World Health Organization classification of overweight and obesity (WHO, 2008). A subject with a BMI value below 18.5 kg m^−2^ was considered to be underweight, a value of 18.5–24.9 kg m^−2^ was regarded as normal, 25–29.9 kg m^−2^ was defined as overweight, and 30 kg m^−2^ and above as obese. Tobacco smoking status was defined as current, former, or never smoker. If the participants had ever smoked ‘one or more cigarettes a day for a year or more’ and ‘smoked within the last 3 months’, they were classified as current smokers. Previous smokers were those who had ever smoked ‘one or more cigarettes a day for a year or more’, but had not smoked during the past 3 months. Never smokers had never smoked ‘one or more cigarettes a day for a year or more’. Formal education was used as a proxy for socioeconomic status, as supported by previous findings (Robert and House, 1996; Fuchs, 2004). Length of education was categorised into less than or equal to 9 years, 10–12 years, or more than 12 years. The use of NSAID was defined as the use of predefined and well-known brands of NSAIDs within the last 3 months. This was categorised into four groups, namely, no use of NSAIDs (or less than once a month), monthly use, weekly use, and daily use, in accordance with previous research (Abnet *et al*, 2009). Aspirin was included in the NSAID variable, as it is considered equivalent to NSAIDs with regard to cancer preventive effects (Abnet *et al*, 2009).

### Statistical analysis

The male and female prevalence rates of reflux, high BMI, tobacco smoking, socioeconomic status, and use of NSAIDs were compared, using exposure frequencies and relative risk estimations. To allow adjustment for potential confounding factors, unconditional multivariable logistic regression was used to calculate odds ratios (ORs) with 95% confidence intervals (CIs). In these analyses, aetiological factors were the exposures and male sex was the outcome, using female sex as reference. Two predefined multivariable models were applied, a basic model adjusted only for age (categorised into three groups: <60, 60–70, or >70 years) and the full model further adjusted for physical activity (several times a week, once a week, or less than once a week), reflux (no or yes), BMI (<25, 25–29.9, or ⩾30 kg m^−2^), tobacco smoking status (never, previous, or current smoker), education (⩽9, 10–12, or >12 years), and NSAID use (ever or never). Physical activity was included as a potential confounder because of reported differences between the sexes and a putative association with the evaluated risk factors (Young *et al*, 2009). Goodness-of-fit (Hosmer and Lemeshow, 1980) was found adequate for both models (data not shown). Furthermore, predefined exploratory analyses were conducted by combining study variables, in which individuals with non-exposure were compared with exposed individuals with regard to given combinations of the included variables. For example, individuals with BMI <25 kg m^−2^, without reflux, who had never smoked were compared with individuals with BMI ⩾25 kg m^−2^, with reflux, who were ever smokers. Intermediate groups of exposure are not presented, but were included in the model, thus using all observations. Owing to the expected small numbers in each category, these analyses were only age-adjusted. Finally, the cutoff of 50 years was used to delineate the presumed effects of menopause, but power was inadequate. Instead, the sample median (65 years) was used to allow age-stratified analyses. All analyses were conducted using STATA 10.1 (StataCorp, College Station, TX, USA).

The Regional Ethics Committee in Stockholm approved the study.

## Results

Among 6969 invited people, the 4906 (70.4%) who responded to the questionnaire were included in this study. Of them, 3220 (65.6%) were men and 1686 (34.4%) were women, with participation rates of 69.5 and 72.6%, respectively. Non-participation was more common in younger age groups; 53.0% of those invited between the ages of 40 and 44 years responded, whereas 75.2% of the ones aged 75–79 years replied. The mean ages of the male and female participants were 65.2 (s.d.=9.4) and 63.9 (s.d.=10.7) years, respectively. The physical activity level was similar in men and women (data not shown). Results from the logistic regression analyses follow, but as the results were similar in the two adjusted models, only the full model is presented.

Reflux was observed in 10.2 and 13.5% of men and women, respectively (Table 1). The adjusted logistic regression analysis identified a statistically significantly lower prevalence of reflux in men than in women (OR=0.70, 95% CI=0.58–0.84; Table 1). Use of the Montreal definition of reflux did not notably alter the sex difference in prevalence (6.2% for men and 8.5% for women).

Among men, 46.8% were overweight and 13.5% were obese, whereas the corresponding prevalence rates for women were 31.9 and 15.1%, respectively (Table 1). After adjustment for other risk factors and other potential confounders in the full regression model, there was an almost two-fold increase in the odds of being overweight in men, as compared with women (OR=1.98, 95% CI=1.72–2.27). The prevalence of obesity was also higher in men, but this sex-associated difference was less marked (OR=1.22, 95% CI=1.01–1.47; Table 1).

The proportion of never smokers was higher among women than among men (52.8 and 45.3%, respectively). Former smoking was more prevalent in men (37.6 *vs* 28.5%), whereas prevalence of current smoking was similar in the two sexes (Table 1). Compared with never smokers, results from the logistic regression analysis suggested that previous smoking, adjusted for all other factors, was 50% more common among men than among women (OR=1.50, 95% CI=1.30–1.72), whereas current smoking was slightly, and non-statistically significantly overrepresented among men (OR=1.18, 95% CI=0.98–1.42; Table 1).

A higher proportion of women than men had more than 12 years of formal education (28.2 *vs* 23.9%). The intermediate educational level (9–12 years) was more common in men than in women (12.3 and 7.1%, respectively). The prevalence of less than 9 years of education was similar between the sexes (Table 1). The adjusted analyses revealed that, compared with the highest education level, the intermediate level was twice as common in men than in women, (OR=2.10, 95% CI=1.65–2.68), whereas the lowest education level was equally distributed between the sexes (OR=1.07, 95% CI=0.92–1.24; Table 1).

Use of NSAIDs was more common among women than among men in all subcategories, and 18.9% of men and 21.1% of women were daily users (Table 1). The adjusted estimates revealed that with daily use as reference, weekly (OR=0.83, 95% CI=0.64–1.06) and monthly (OR=0.89, 95% CI=0.68–1.15) use was non-significantly less common in men than in women. Furthermore, non-use of NSAIDs was more prevalent in men (OR=1.35, 95% CI=1.14–1.59; Table 1).

Simultaneous exposures to combinations of some or all study variables are shown in Table 2. A marked male predominance was observed in combined exposure to reflux, high BMI, and NSAID use (OR=1.62, 95% CI=1.09–2.42), the combination of reflux, high BMI, tobacco smoking, and NSAID use (OR=2.59, 95% CI=1.42–4.72), and the combined exposure to all five studied factors (OR=2.76, 95% CI=1.21–6.32). Evaluated associations of male sex with other combinations of risk factors were not statistically significant (Table 2).

Stratifying for age by using the sample median of 65 years produced logistic regression results as shown in Table 3. Overweight (OR=2.41, 95% CI=1.99–2.93 *vs* OR=1.63, 95% CI=1.33–1.99) and obesity (OR=1.74, 95% CI=1.33–2.29 *vs* OR=0.87, 95% CI=0.67–1.13) seemed to be more associated with male sex compared with female sex at younger ages. Previous (OR=1.04, 95% CI=0.86–1.27 *vs* OR=2.18, 95% CI=1.79–2.67) and current (OR=0.97, 95% CI=0.76–1.24 *vs* OR=1.41, 95% CI=1.05–1.90) smoking was less strongly linked to men at younger ages than to men at older ages. Younger men seemed to have a shorter education than younger women (⩽9 years: OR=1.38, 95% CI=1.13–1.67), whereas this association was reversed at older age (⩽9 years: OR=0.77, 95% CI=0.61–0.98). Finally, no use of NSAID seemed equally more prevalent in younger and older men (OR=1.26, 95% CI=0.95–1.67 *vs* OR=1.31, 95% CI=1.07–1.61; Table 3).

## Discussion

This study of a random sample of the general population indicates that exposure to risk factors for oesophageal adenocarcinoma is more common among men than among women. There was no male predominance regarding reflux alone, but each of the risk factors, namely, high BMI, tobacco smoking, and low socioeconomic status, was more common among men and use of NSAIDs was less prevalent among men. Combinations of these risk factors were more prevalent in men only when use of NSAID was included. Age-stratified analyses indicated that high BMI was more common in men at a younger age.

The advantages of this study include a population-based design with a high participation rate, which reduces the risk of selection bias and facilitates generalisation. Moreover, the large sample size allowed robust estimations, combining of study variables and stratification. The availability of data on all known risk factors allowed adjustment for potential confounding. There are, however, several limitations. The use of questionnaires to evaluate variables, such as height and weight, could introduce misclassification, and women might underestimate weight and overestimate height more than men (Flood *et al*, 2000). This effect, however, is mostly mediated by socioeconomic differences (Bostrom and Diderichsen, 1997), for which adjustment was made in this study. Missing values could introduce biased results, but the extent of missing data was limited and any such effect should be non-differential and therefore not explain the positive associations. Risk factors for cancer development are commonly considered to have an impact over a number of years. It might therefore be argued that a younger population sample should have been chosen to reflect the risk factors when cancer development was initiated. However, the induction times for the mechanisms that cause oesophageal adenocarcinoma are not known, and habits already established in adulthood may not readily be prone to change (Prattala *et al*, 1994; Mulder *et al*, 1998; Benzies *et al*, 2008). Residual confounding from known risk factors and confounding from unknown variables are threats to all observational studies. However, adjustments were made for all known risk factors and the categorisation was comparatively detailed. Some data suggest that infection with *Helicobacter pylori* prevents the development of oesophageal adenocarcinoma (Rokkas *et al*, 2007), but this possible negative association remains to be established. Moreover, data regarding a possible sex difference in *H. pylori* prevalence are conflicting, at most showing a weak male predominance of infection in adults (de Martel and Parsonnet, 2006). Multiple testing is another issue to be considered in this study, as several analyses were conducted and we combined various risk factors; however, this concern should be mitigated by the fact that the hypotheses were predefined and the exploratory evaluation of the combination of variables was planned before the initiation of any analysis.

In previous studies, the sex-specific prevalence rates of the five known risk factors have been evaluated separately. The reflux prevalence has not been observed to be higher in males (Locke *et al*, 1997; Nilsson *et al*, 2004), an observation confirmed by this study. Findings of the national surveys of BMI in the United States (Ogden *et al*, 2006) and Europe (Andreyeva *et al*, 2007) are consistent with the prevalence pattern observed in this study, that is, a higher BMI in men. Our finding of a lower frequency of non-smoking in females is in line with most previous reports (CDC, 2007), although the prevalence rates in Sweden, especially at younger ages, have more recently been observed to be higher in women (Ali *et al*, 2009). In our study, women had, on an average, a longer education, whereas previous research indicated a more similar sex distribution regarding the number of years of formal education (Molarius *et al*, 2007). The use of NSAIDs and aspirin was more common in women than in men, which is supported by some previous data with regard to aspirin (Larsson *et al*, 2006). Thus, the external validity of our study seems to be adequate. Indeed, the result that reflux seems to be less common in men might be a product of the comparatively high average age of participants in this study, as reflux prevalence in older men as compared with older women has previously been shown to be lower (Locke *et al*, 1997; Nilsson *et al*, 2004). Disregarding reflux, the multivariable analysis revealed small but significant differences in separate risk factor exposure, favouring an increased exposure in men. Putting these results into perspective, population attributable risks were calculated (Levin, 1953; Taylor, 1977) for the main exposures, namely, reflux, high BMI, and tobacco smoking, using another Swedish database, incorporating oesophageal adenocarcinoma data (Lofdahl *et al*, 2008). For the presence of reflux disease, a BMI value >25 kg m^−2^, and ever smoking, these attributable risks were 20, 40, and 43% for men and 33, 45, and 49% for women, respectively.

The finding that a combination of the two main risk factors, reflux disease and high BMI, even when smoking status was included, did not result in significant associations with male sex, warrants a comment. The absence of clustering of these risk factors in men might be explained by women reporting more reflux, although overweight and ever smoking were more frequent in men. The present study was unable to take into account different types of overweight and reflux; for example, the predominantly abdominal type of obesity (Corley *et al*, 2008) and erosive reflux disease (Cook *et al*, 2005) are more common in males than in females, and both these exposures have been shown to be more harmful with regard to carcinogenesis (Cook *et al*, 2005).

When combining all risk factors to evaluate clustering, the data in this study indicate predominance in men, which seems to be driven mainly by differential NSAID use in men and women, a comparatively weak and uncertain aetiological factor for this cancer, without which no significant difference could be discerned. There is mounting evidence that the male predominance in oesophageal adenocarcinoma is age specific, wherein the highest incidence rate ratios are observed at younger ages (Cook *et al*, 2009; Derakhshan *et al*, 2009; Rutegard *et al*, 2010). This may reflect changes during and after menopause, and therefore the cutoff of 50 years was attempted for stratification. However, the sample size only allowed the use of the sample median of 65 years, which may not be entirely appropriate from a biological perspective. Nevertheless, it seems that a high BMI value is even more prevalent in men at younger age. This is intriguing, particularly as some evidence indicates that this high BMI value in men confers higher risks of oesophageal adenocarcinoma compared with women (Ryan *et al*, 2006).

This first and large population-based study with an unselected sampling of participants indicates that exposure to some, but not all, established risk factors for oesophageal adenocarcinoma is overrepresented in males compared with females. The male preponderance to simultaneous exposure to all risk factors is mainly due to differential NSAID use in men and women. Our findings seem unlikely to explain the male predominance.

## Figures and Tables

**Table 1:** Sex-specific prevalence rates and results of logistic regression analyses

	**Men: *N*=3220 (65.6%)**	**Women: *N*=1686 (34.4%)**	**Full model** a
**Selected risk factors**	***N* (%)**	***N* (%)**	**OR (95% CI)**
*Reflux* b			
No	2711 (84.2)	1301 (77.2)	1.00 (reference)
Yes	330 (10.2)	227 (13.5)	0.70 (0.58–0.84)
*Missing*	*179* (*5.6)*	*158* (*9.4)*	
			
*Body Mass Index*			
<25 (normal weight)	1120 (34.8)	790 (46.9)	1.00 (reference)
25–30 (overweight)	1508 (46.8)	538 (31.9)	1.98 (1.72–2.27)
⩾30 (obese)	435 (13.5)	254 (15.1)	1.22 (1.01–1.47)
* Missing*	*157* (*4.9)*	*104* (*6.2)*	
			
*Tobacco smoking status*			
Never smoker	1459 (45.3)	890 (52.8)	1.00 (reference)
Former smoker	1210 (37.6)	480 (28.5)	1.50 (1.30–1.72)
Current smoker	445 (13.8)	233 (13.8)	1.18 (0.98–1.42)
*Missing*	*106* (*3.3)*	*83* (*4.9)*	
			
*Formal education (proxy for SES)*			
>12 years	771 (23.9)	475 (28.2)	1.00 (reference)
9–12 years	397 (12.3)	119 (7.1)	2.10 (1.65–2.68)
⩽9 years	1953 (60.7)	1034 (61.3)	1.07 (0.92–1.24)
*Missing*	*99* (*3.1)*	*58* (*3.4)*	
			
*NSAID use*			
Daily	608 (18.9)	356 (21.1)	1.00 (reference)
Weekly	216 (6.7)	170 (10.1)	0.83 (0.64–1.06)
Monthly	217 (6.7)	156 (9.3)	0.89 (0.68–1.15)
No use^c^	2023 (62.8)	926 (54.9)	1.35 (1.14–1.59)
*Missing*	*156* (*4.8)*	*78* (*4.6)*	
			

Abbreviations: CI=confidence interval; N=number; NSAID=non-steroidal anti-inflammatory drug; OR=odds ratio; SES=socioeconomic status.

Sex-specific prevalence rates and results of logistic regression analyses with OR and 95% CIs values in a randomly selected sample of 4906 Swedish citizens, using risk factors as exposures and male sex as outcome.

a Adjusted for age, physical activity, reflux, education, body mass index, smoking status, NSAID use.

b Defined as at least weekly symptoms of acid regurgitation and/or heartburn and/or weekly use of gastro-oesophageal reflux disease treatment, such as proton pump inhibitors, antacids, or H2-blockers.

c No use or less than once a month.

**Table 2:** Sex-specific prevalence rates and results of logistic regression analyses

	**Men: *N*=3220 (65.6%)**	**Women: *N*=1686 (34.4%)**	**Logistic model** a
**Risk factor combination**	***N* (%)**	***N* (%)**	**OR (95% CI)**
*Reflux*^b^ *and BMI (kg m*^−*2*^)			
Reflux-negative, BMI<25	965 (30.0)	645 (38.3)	1.00 (reference)
Reflux-positive, BMI⩾25	211 (6.6)	139 (8.2)	0.98 (0.77–1.24)
			
*Reflux*^b^, *BMI (kg m*^−*2*^), *and smoking*			
Reflux-negative, BMI<25, never smoker	482 (15.0)	334 (19.8)	1.00 (reference)
Reflux-positive, BMI⩾25, ever smoker	117 (3.6)	62 (3.7)	1.27 (0.90–1.78)
			
*Reflux*^b^, *BMI (kg m*^−*2*^), *and SES*			
Reflux-negative, BMI<25, education >9 years	424 (13.2)	293 (17.4)	1.00 (reference)
Reflux-positive, BMI⩾25, education⩽9 years	131 (4.1)	88 (5.2)	0.90 (0.66–1.24)
			
*Reflux*^b^, *BMI (kg m*^−*2*^), *and NSAIDs*			
Reflux-negative, BMI<25, ever use of NSAIDs	267 (8.3)	204 (12.1)	1.00 (reference)
Reflux-positive, BMI⩾25, never use of NSAIDs	97 (3.0)	45 (2.7)	1.62 (1.09–2.42)
			
*Reflux*^b^, *BMI (kg m*^−*2*^), *smoking*, *and SES*			
Reflux-negative, BMI<25, never smoker, education >9 years	244 (7.6)	160 (9.5)	1.00 (reference)
Reflux-positive, BMI⩾25, ever smoker, education ⩽9 years	72 (2.2)	36 (2.1)	1.17 (0.74–1.83)
			
*Reflux*^b^, *BMI (kg m*^−*2*^), *smoking*, *and NSAIDs*			
Reflux-negative, BMI<25, never smoker, ever use of NSAIDs	120 (3.7)	113 (6.7)	1.00 (reference)
Reflux-positive, BMI⩾25, ever smoker, never use of NSAIDs	50 (1.6)	18 (1.1)	2.59 (1.42–4.72)
			
*Reflux*^b^, *BMI (kg m*^−*2*^), *SES*, *and NSAIDs*			
Reflux-negative, BMI<25, education >9 years, ever use of NSAIDs	95 (3.0)	86 (5.1)	1.00 (reference)
Reflux-positive, BMI⩾25, education ⩽9 years, never use of NSAIDs	60 (1.9)	27 (1.6)	1.79 (1.04–3.10)
			
*Reflux*^b^, *BMI (kg m*^−*2*^*), smoking*, *SES*, *and NSAIDs*			
Reflux-negative, BMI<25, education >9 years, ever use of NSAIDs, never smoker	47 (1.5)	47 (2.8)	1.00 (reference)
Reflux-positive, BMI⩾25, education ⩽9 years, never use of NSAIDs, ever smoker	31 (1.0)	10 (0.6)	2.76 (1.21–6.32)
			

Abbreviations: BMI=body mass index; CI=confidence interval; N=number; NSAID=non-steroidal anti-inflammatory drug; OR=odds ratio; SES=socioeconomic status.

Sex-specific prevalence rates and results of logistic regression analyses with OR and 95% CIs values, in a randomly selected sample of 4906 Swedish citizens, using predefined combinations of risk factors as exposures and male sex as outcome.

a Using male sex as outcome and adjusted for age only.

b Gastro-oesophageal reflux disease, defined as at least weekly symptoms of acid regurgitation and/or heartburn and/or weekly use of reflux treatment such as proton pump inhibitors, antacids, H2-blockers, etc.

**Table 3:** Age-stratified sex-specific prevalence rates and results of logistic regression analyses

	**Age⩽65 years**			**Age >65 years**		
	**Men: *N*=1519 (63.7%)**	**Women: *N*=865 (36.3%)**	**Full model** a	**Men: *N*=1701 (67.4%)**	**Women: *N*=821 (32.6%)**	**Full model** a
**Selected risk factors**	***N* (%)**	***N* (%)**	**OR (95% CI)**	***N* (%)**	***N* (%)**	**OR (95% CI)**
*Reflux* b						
No	1319 (86.8)	716 (82.8)	1.00 (reference)	1392 (81.8)	585 (71.3)	1.00 (reference)
Yes	147 (9.7)	112 (12.9)	0.70 (0.53–0.92)	183 (10.8)	115 (14.0)	0.69 (0.53–0.90)
						
*Body Mass Index*						
<25 (normal weight)	513 (33.8)	453 (52.4)	1.00 (reference)	607 (35.7)	337 (41.0)	1.00 (reference)
25–30 (overweight)	734 (48.3)	269 (31.1)	2.41 (1.99–2.93)	774 (45.5)	269 (32.8)	1.63 (1.33–1.99)
⩾30 (obese)	219 (14.4)	110 (12.7)	1.74 (1.33–2.29)	216 (12.7)	144 (17.5)	0.87 (0.67–1.13)
						
*Tobacco smoking status*						
Never	700 (46.1)	405 (46.8)	1.00 (reference)	759 (44.6)	485 (59.1)	1.00 (reference)
Former smoker	522 (34.4)	281 (32.5)	1.04 (0.86–1.27)	688 (40.4)	199 (24.2)	2.18 (1.79–2.67)
Current smoker	265 (17.4)	154 (17.8)	0.97 (0.76–1.24)	180 (10.6)	79 (9.6)	1.41 (1.05–1.90)
						
*Formal education (proxy for SES)*						
>12 years	460 (30.3)	356 (41.2)	1.00 (reference)	311 (18.3)	119 (14.5)	1.00 (reference)
9–12 years	249 (16.4)	89 (10.3)	2.10 (1.58–2.80)	148 (8.7)	30 (3.7)	2.14 (1.35–3.37)
⩽9 years	771 (50.8)	399 (46.1)	1.38 (1.13–1.67)	1182 (69.5)	635 (77.3)	0.77 (0.61–0.98)
						
*NSAID use*						
Daily	608 (18.9)	356 (21.1)	1.00 (reference)	441 (25.9)	256 (31.2)	1.00 (reference)
Weekly	216 (6.7)	170 (10.1)	0.71 (0.48–1.05)	110 (6.5)	76 (9.3)	0.85 (0.61–1.21)
Monthly	217 (6.7)	156 (9.3)	0.71 (0.49–1.02)	79 (4.6)	37 (4.5)	1.15 (0.74–1.78)
No use^c^	2023 (62.8)	926 (54.9)	1.26 (0.95–1.67)	946 (55.6)	388 (47.3)	1.31 (1.07–1.61)
						

Abbreviations: CI=confidence interval; N=number; NSAID=non-steroidal anti-inflammatory drug; OR=odds ratio; SES=socioeconomic status.

Age-stratified sex-specific prevalence rates and results of logistic regression analyses OR and 95% CIs values, in a randomly selected sample of 4906 Swedish citizens, using predefined combinations of risk factors as exposures and male sex as outcome.

a Adjusted for physical activity, reflux, education, body mass index, smoking status, NSAID use.

b Defined as at least weekly symptoms of acid regurgitation and/or heartburn and/or weekly use of gastro-oesophageal reflux disease treatment such as proton pump inhibitors, antacids, or H2-blockers.

c No use or less than once a month.
